# Magnetic Activated Carbon from ZnCl_2_ and FeCl_3_ Coactivation of Lotus Seedpod: One-Pot Preparation, Characterization, and Catalytic Activity towards Robust Degradation of Acid Orange 10

**DOI:** 10.1155/2023/3848456

**Published:** 2023-06-06

**Authors:** Dung Van Nguyen, Hung Minh Nguyen, Quang Le Nam Bui, Thao Vy Thanh Do, Hung Hoa Lam, Tuyet-Mai Tran-Thuy, Long Quang Nguyen

**Affiliations:** ^1^Faculty of Chemical Engineering, Ho Chi Minh City University of Technology (HCMUT), 268 Ly Thuong Kiet Street, District 10, Ho Chi Minh City, Vietnam; ^2^Vietnam National University Ho Chi Minh City, Linh Trung Ward, Thu Duc City, Ho Chi Minh City, Vietnam

## Abstract

Lotus seedpods (LSPs) are an abundant and underutilized agricultural residue discarded from lotus seed production. In this study, ZnCl_2_ and FeCl_3_ coactivation of LSP for one-pot preparation of magnetic activated carbon (MAC) was explored for the first time. X-ray diffraction **(**XRD) results showed that Fe_3_O_4_, Fe^0^, and ZnO crystals were formed in the LSP-derived carbon matrix. Notably, transmission electron microscopy **(**TEM) images showed that the shapes of these components consisted of not only nanoparticles but also nanowires. Fe and Zn contents in MAC determined by atomic absorption spectroscopy **(**AAS) were 6.89 and 3.94 wt%, respectively. Moreover, *S*_BET_ and *V*_total_ of MAC prepared by coactivation with ZnCl_2_ and FeCl_3_ were 1080 m^2^/g and 0.51 cm^3^/g, which were much higher than those prepared by single activation with FeCl_3_ (274 m^2^/g and 0.14 cm^3^/g) or ZnCl_2_ (369 m^2^/g and 0.21 cm^3^/g). MAC was subsequently applied as an oxidation catalyst for Fenton-like degradation of acid orange 10 (AO10). As a result, 0.20 g/L MAC could partially remove AO10 (100 ppm) with an adsorption capacity of 78.4 mg/g at pH 3.0. When 350 ppm H_2_O_2_ was further added, AO10 was decolorized rapidly, nearly complete within 30 min, and 66% of the COD was removed in 120 min. The potent catalytic performance of MAC might come from the synergistic effect of Fe^0^ and Fe_3_O_4_ nanocrystals in the porous carbon support. MAC also demonstrated effective stability and reusability after five consecutive cycles, when total AO10 removal at 20 min of H_**2**_O_**2**_ addition slightly decreased from 93.9 ± 0.9% to 86.3 ± 0.8% and minimal iron leaching of 1.14 to 1.19 mg/L was detected. Interestingly, the MAC catalyst with a saturation magnetization of 3.6 emu/g was easily separated from the treated mixture for the next cycle. Overall, these findings demonstrate that magnetic activated carbon prepared from ZnCl_2_ and FeCl_3_ coactivation of lotus seedpod waste can be a low-cost catalyst for rapid degradation of acid orange 10.

## 1. Introduction

Today, agricultural activities generate enormous amounts of solid wastes all over the world. These agricultural wastes are commonly disposed of by burning them in the fields. This activity can cause a variety of ecological and environmental problems [1]. Hence, numerous studies on the valorization of agricultural residues have been done in light of different economic, energy, and environmental concerns [2, 3]. Typically, agricultural wastes consist of lignocellulosic biomass, which includes cellulose, hemicellulose, and lignin [4]. Due to their carbon resources, these wastes can be employed in the production of carbon-based materials [5, 6].

Biochar (BC) is a carbon-rich material prepared from the pyrolysis of different biomass resources in oxygen-free environments [7–11]. Despite the vast variety of carbon-based materials, BC is an inexpensive, readily available, and convertible material [12, 13]. Moreover, it possesses advantageous physicochemical features, porous structures, and varied functional groups [14]. Thus, BC is widely utilized for gas storage and separation, soil treatment, wastewater treatment, electrodes, and energy storage [15–18]. Regardless of this, it is challenging to separate BC from its suspension [19, 20]. Traditional separation techniques are often expensive or insufficient, thereby severely limiting the application of BC [21]. Consequently, introducing magnetic components into BC can overcome this disadvantage. Different magnetic components such as Fe^0^, Fe_2_O_3_, Fe_3_O_4_, and MnFe_2_O_4_ particles could be dispersed on BC, resulting in a new material known as magnetic biochar (MBC).

To synthesize MBC from biomass, magnetic precursors are commonly loaded onto carbon surfaces [22]. This old approach, however, is not only complex, but it also closes existing pores in carbon supports [23]. In recent years, a growing number of publications focusing on producing MBC using one-pot pyrolysis of magnetic precursor-loaded biomass have been developed [24, 25]. It involves directly dispersing magnetic precursors like FeCl_3_ into biomass resources and then pyrolyzing the obtained mixtures to yield MBC [26, 27]. According to Bedia et al. [28], biomass activated with FeCl_3_ produces MBC with well-dispersed iron-based nanoparticles and well-developed porosity. However, compared with other well-known activating agents, FeCl_3_ has limited activation efficiency. Porous systems of MBC grow slightly. For instance, specific surface areas (*S*_BET_) of MBC are obtained from municipal sludge (FeCl_3_/N_2_): 38 m^2^/g [29]; spent coffee grounds (FeCl_3_/N_2_): 8 m^2^/g [30]; peanut hull (FeCl_3_/N_2_): 159 m^2^/g [31]; and lotus stem (FeCl_3_/O_2_-limited): 374 m^2^/g [32]. Increasing the FeCl_3_/biomass ratio could improve the activation process. However, an excessive ratio can affect the porous properties and application performance of MBC [23, 33]. FeCl_3_ should be well impregnated inside the natural holes of biomass. Therefore, high FeCl_3_ loading may form bigger Fe-based particles and clusters, decreasing the surface area of catalytic Fe sites. The bigger Fe-based particles may also block the pores of carbon bases, negatively affecting mass transfer. The interaction between MBC and organic pollutants mainly comes from surface porous carbon with functional groups rather than Fe-based particles. High Fe loading content may decrease adsorption sites, causing weaker adsorption. To expand the porous system of MBC effectively, a few recent reports propose the combination of FeCl_3_ with another activating agent during one-pot pyrolysis of biomass. By replacing N_2_ with CO_2_, the *S*_BET_ of MBC obtained from the FeCl_3_-activation of spent coffee grounds increased remarkably from 8 to 512 m^2^/g [30]. Hence, the additional activation during one-pot preparation of MBC is necessary, and the resulting material can be referred to as magnetic activated carbon (MAC).

Physical and chemical activation are the two most common techniques used to activate carbon-based materials [12]. Physical activation is a two-step technique that first produces activated carbon by carbonizing biomass and then activates it at high temperatures with H_2_O or CO_2_ [34, 35]. For chemical activation, biomass resources are first impregnated with activating agents such as KOH, K_2_CO_3_, H_3_PO_4_, H_2_SO_4_, AlCl_3_, ZnCl_2_, and FeCl_3_, and then carbonized and activated in one-step pyrolysis. Those agents might theoretically activate MBC [36, 37]. The selection of an effective approach for expanding a porous system should not, however, impact the magnetic and other properties of the original MBC. Physical activation must be conducted within a range of high temperatures and high pressures, resulting in the potential for severe changes in the different properties of MAC products [34, 35]. Therefore, chemical activation with a powerful activating agent like ZnCl_2_ is preferable [38, 39]. Experiments revealed that ZnCl_2_-activated carbon possessed a greater surface area and a greater number of micropores. In addition, the aromatic structure of ZnCl_2_-activated carbon was enhanced [40]. Hence, it is crucial to activate biomass with the combination of ZnCl_2_ and FeCl_3_ when few studies have been found in the literature. Lee and Ahmad Zaini [41] demonstrated that ZnCl_2_ and FeCl_3_ coactivation of palm kernel shell offered MAC with a very high *S*_BET_ of 1775 m^2^/g. As a result, the obtained MAC demonstrated exceptional adsorption of rhodamine B at 371 mg/g. Similarly, Lou et al. [42] prepared MAC from corn stover with a *S*_BET_ of 1409 m^2^/g for significantly enhanced Cr (VI) removal of 185.8 mg/g. Based on the abovementioned results, it is possible to use both ZnCl_2_ and FeCl_3_ as an activating mixture in a facile one-pot preparation of MAC from biomass resources. In terms of adsorption, the superiority of MAC over MBC has been proven; however, its catalytic activity has been studied very little. Such reports [21, 43] indicated that Fe-based particles in MBC could become catalytic sites for effective treatment of organic compounds through advanced oxidation processes. Thus, it is anticipated that MAC with Fe-based sites and an expanded porous system could exhibit better catalytic performance. To increase the possible use of MAC, its catalytic performance must be investigated in greater depth.

Nowadays, numerous industrial processes, including food processing, papermaking, printing, leather, textiles, cosmetics, and pharmaceuticals, discharge vast quantities of dyes into the aquatic environment [44–46]. Dye pollution is a significant environmental concern because synthetic dyes are typically not biodegradable, meaning they persist for extended periods of time in the environment [47, 48]. In addition to causing aesthetic issues, dyes can impair the survival and reproduction of aquatic organisms [49–51]. Hence, effective remediation of dye pollution in wastewater is essential for environmental protection and sustainable development. Biodegradation, adsorption, coagulation-flocculation, photocatalytic treatment, and chemical treatment are typical techniques [52–55]. With chemical treatment, dye molecules can be oxidized and broken down using chemicals, such as hydrogen peroxide, ozone, and persulfate, rendering them less toxic and simpler to remove from the environment [56]. Hydrogen peroxide (H_2_O_2_) offers several advantages over other oxidizing agents, including its safety, environmental friendliness, versatility, cost-effectiveness, simple operation, and mild conditions [57, 58]. However, the use of H_2_O_2_ alone is less effective. To accelerate the treatment of organic pollutants by H_2_O_2_, catalysts can be used. As mentioned before, magnetic biochar has proven that it is an effective catalyst for the treatment of synthetic dyes by H_2_O_2_ owing to its effectiveness, stability, low cost, and environmental friendliness [27, 59].

Lotus seedpods (LSP) are released from seed gathering in markets and factories, resulting in massive agricultural waste [60, 61]. LSP is a prospective carbon resource for the production of various carbon-based products on account of its availability, abundance, underutilization, and low cost. In our previous studies, LSP was used to prepare MBC through one-pot FeCl_3_ activation [19, 27]. The developed MBCs exhibited efficient catalytic activity for the elimination of organic contaminants by H_2_O_2_. Herein, LSP was continuously selected as a biomass resource for one-pot preparation of MAC using ZnCl_2_ and FeCl_3_ coactivation. To evaluate the catalytic performance of as-prepared MAC samples in a Fenton-like process, acid orange 10 (AO10), a synthetic azo dye with extensive usage, limited biodegradability, and potential toxicity [62–64] was selected.

## 2. Materials and Methods

### 2.1. Materials

Raw lotus seedpod residue was received from a factory for lotus seed production located in Thap Muoi District, Dong Thap Province, Vietnam. The pods were washed with tap and distilled water to remove all dirt before being dried in an electric drying oven at 105°C for 24 h. Then, the raw material was cut and milled to obtain a fine powder. To avoid moisture, the powder was stored in an airtight vessel for later use. FeCl_3_.6H_2_O (≥99.0%), ZnCl_2_ (≥98.0%), H_2_SO_4_ (95.0–98.9%), NaOH (≥96.0%), Na_2_S_2_O_3_.5H_2_O (≥99.0%), H_2_O_2_ (≥30.0%), KH_2_PO_4_ (≥99.5%), Na_2_HPO_4_.12H_2_O (≥99.0%), and acid orange 10 were obtained from Xilong Scientific Co., Ltd., China. All analytical grade chemicals were used directly, without further refinement.

### 2.2. Preparation of Magnetic Activated Carbon from Lotus Seedpod

Magnetic activated carbon was prepared via the one-pot pyrolysis of ZnCl_2_ and FeCl_3_-loaded lotus seedpod residue. First, 4.00 g of LSP powder, 0.80 g of FeCl_3_, and a certain amount (4x *g*) of ZnCl_2_ were added to 100 mL of distilled water. After 3.0 h of stirring, the mixture was dried in an oven at 105°C for 24 h. The dried sample was then added to a glass reaction tube in a vertical furnace. A constant nitrogen flow rate of 250 mL/min maintained the inert atmosphere inside the tube. To pyrolyze, the tube was heated from room temperature to 600°C at an average rate of 5°C/min and then held at that temperature for 60 min. The obtained solid was washed repeatedly to remove all residual FeCl_3_ and ZnCl_2_. Wastewater was tested with a pH meter, an electrical conductivity meter, an aqueous NaOH solution, and an aqueous AgNO_3_ solution to detect ion leaching (Fe^3+^, Zn^2+^, and Cl^−^). Lastly, the sample was dried at 80°C for 24 h to obtain MAC. Due to the mass ratio of ZnCl_2_/FeCl_3_/LSP being x/0.2/1.0, the as-prepared MAC samples were denoted as MAC-x. Moreover, biochar (BC), magnetic biochar (MBC), and activated carbon (ZAC), which served as reference samples, were prepared by the pyrolysis of LSP, FeCl_3_-loaded LSP, and ZnCl_2_-loaded LSP under the same procedure. These labels are presented in Table 1.

### 2.3. Characterization of Magnetic Activated Carbon

Powder X-ray diffraction (XRD) in the 2*θ* = 10–80° range was measured on a Bruker AXS D8 diffractometer using CuK *α* radiation (*λ* = 1.5418 Å). Fe and Zn contents in MBC and MAC samples were analyzed by a Perkin Elmer Analyst 800 atomic absorption spectrophotometer (AAS). These metal elements were extracted from MBC and MAC samples in a HCl (6 M) solution at 60°C for 60 min. Nitrogen adsorption and desorption isotherms of MBC, ZAC, and MAC were measured at 77 K on a Micromeritics® TriStar II Plus. All samples were degassed at 250°C for 5 h. The specific surface area (*S*_BET_) was calculated from the Brunauer–Emmett–Teller equation. The total pore volume (*V*_total_) was determined at *P*/*P*_o_ = 0.995. The average pore size (*d*_average_) was obtained from 4*V*_total_/*S*_BET_. The pore size distribution was determined by the BJH method. The magnetic properties of MBC and MAC were examined with a vibrating sample magnetometer (VSM) at room temperature. Fourier transform infrared (FTIR) spectroscopy of MAC was performed using a Tensor 27 spectrometer. Scanning electron microscope (SEM) images, energy dispersive X-ray (EDX) spectroscopy, and elemental mapping of MAC were analyzed using a JEOL JSM-IT200 instrument. Transmission electron microscopy (TEM) images of BC, MBC, and MAC were recorded by a JEOL JEM-1010 instrument.

### 2.4. Degradation of Acid Orange 10 Using Magnetic Activated Carbon

The catalytic performance of MAC samples was explored through the degradation of acid orange 10 using H_2_O_2_ as an oxidizing agent at room temperature (30°C). In brief, 500 mL of AO10 (100 ppm) and a certain MAC dosage were added to a 1000 mL glass cylinder. The initial pH value of the mixture was adjusted using H_2_SO_4_ (0.5 M) and NaOH (0.1 M) solutions. The adsorption step was carried out within the first 20 min. The adsorption capacity (Q), therefore, was calculated from the following equation:

(1) $$\begin{array}{c} Q\left(\frac{mg}{g}\right)=\frac{C_{0}^{A}-C_{20}^{A}}{C_{M}}\text{,} \end{array}$$

where *C*_*M*_ (g/L) is the material dosage, and *C*_0_^*A*^ and *C*_20_^*A*^ (ppm) are the AO10 concentrations at the beginning and after 20 min of adsorption.

After the adsorption step, the oxidation step was initiated by the rapid addition of H_2_O_2_ to the mixture. Samples taken were added immediately to a solution of phosphate buffer and Na_2_S_2_O_3_ (2.0 g/L) to adjust the pH to 7.0 and eliminate excess H_2_O_2_. AO10 concentrations were quantitatively examined at 480 nm with a UV-Vis spectrophotometer (Lovibond PC Spectro). The decolorization efficiency and total removal of AO10 were calculated as follows:

(2) $$\begin{array}{c} Decolorization\;efficiency\left(\%\right)=\frac{C_{0}^{O}-C_{30}^{O}}{C_{0}^{O}}\times 100\%\text{,}\\ Total\;removal\left(\%\right)=\frac{C_{0}^{A}-C_{30}^{O}}{C_{0}^{A}}\times 100\%\text{,} \end{array}$$

where *C*_0_^*O*^ and *C*_30_^*O*^ (ppm) are the AO10 concentrations at the beginning and after 30 min of oxidation.

The chemical oxidation demand (COD) was quantified using the closed-reflux titrimetric method (5220C) [65]. To minimize the influence of residual H_2_O_2_ on COD results, samples were mixed with a solution of 20.0 g/L Na_2_CO_3_ and incubated at 90°C for 60 min [66].

To evaluate the stability and reusability of the MAC catalyst, a sample was used in five consecutive experiments. The used catalyst was recovered using a magnet, rinsed with distilled water and ethanol, and then placed in an oven at 110°C. The dried catalyst was weighed in preparation for the subsequent experiment. At the end of each cycle, the treated solution was analyzed with the previously mentioned AAS instrument to identify Fe leaching.

## 3. Results and Discussion

### 3.1. Characterization of Magnetic Activated Carbon

#### 3.1.1. XRD Patterns of MBC, ZAC, and MAC

**Figure 1:**
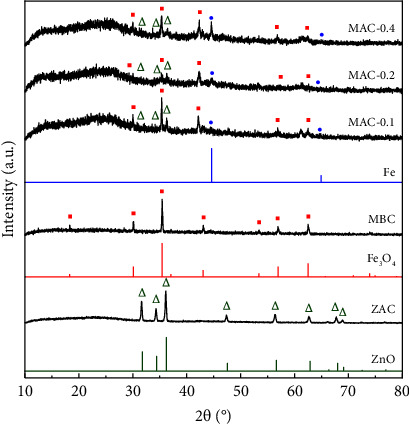
XRD patterns of MBC, ZAC, and MAC samples.

XRD was used to examine the development of crystals on MBC, ZAC, and MAC samples, as shown in Figure 1. Peaks of Fe_3_O_4_ crystals were found in MBC at 2*θ* = 18.3, 30.1, 35.4, 42.4, 52.4, 56.0, and 61.5°, respectively, corresponding to the (111), (220), (311), (400), (422), (511), and (440) planes (JCPDS 19-0629). The following reactions are proposed for the formation of Fe_3_O_4_ during the one-pot pyrolysis of FeCl_3_-loaded LSP:

(3) $$\begin{array}{c} Lotus\;seedpod\;⟶\;H_{2}O,H_{2},CO,C \end{array}$$

(4) $$\begin{array}{c} {FeCl}_{3}+3H_{2}O\;⟶\;{Fe\left(OH\right)}_{3}+3HCl \end{array}$$

(5) $$\begin{array}{c} {2Fe\left(OH\right)}_{3}\;⟶\;{{Fe}_{2}O}_{3}+{3H}_{2}O \end{array}$$

(6) $$\begin{array}{c} {{3Fe}_{2}O}_{3}+CO\;⟶\;{{2Fe}_{3}O}_{4}+CO_{2} \end{array}$$

(7) $$\begin{array}{c} {{3Fe}_{2}O}_{3}+H_{2}\;⟶\;{{2Fe}_{3}O}_{4}+H_{2}O \end{array}$$

(8) $$\begin{array}{c} {{3Fe}_{2}O}_{3}+C\;⟶\;{{2Fe}_{3}O}_{4}+CO \end{array}$$

(9) $$\begin{array}{c} {{Fe}_{3}O}_{4}+4C\;⟶\;3Fe+4CO \end{array}$$

With ZnCl_2_ activation, ZAC possessed the peaks at 2*θ* = 31.7, 34.3, 36.1, 47.4, 56.3, 62.7, 67.7, and 68.9°, which respectively correspond to the (100), (002), (101), (102), (110), (103), (112), and (201) planes of hexagonal ZnO crystals (JCPDS 36-1451). Based on a report by Ma [40], the following equations might explain the production of ZnO:

(10) $$\begin{array}{c} Lotus\;seedpod\;\left(C_{x}H_{y}O_{z}\right)+2ZnCl_{2}\;⟶C_{x}H_{y-6}O_{z-3}+Zn_{2}O{Cl}_{2}.2H_{2}O+2HCl \end{array}$$

(11) $$\begin{array}{c} Zn_{2}O{Cl}_{2}.2H_{2}O\;⟶\;ZnCl_{2}+ZnO+2H_{2}O \end{array}$$

At high temperatures, molten ZnCl_2_ can promote dehydration processes to cleave polymer chains of lignocellulosic biomass, yielding H_2_O and a thermoplastic carbonaceous phase [67]. ZnCl_2_ can then combine with H_2_O to produce Zn_2_OCl_2_.2H_2_O. Subsequently, the decomposition of Zn_2_OCl_2_.2H_2_O can produce ZnCl_2_ vapor, and its diffusion can activate the thermoplastic phase to offer the last porous carbon system [68]. Moreover, the formed ZnO can be kept in the carbon structure.

By coactivation of LSP with ZnCl_2_ and FeCl_3_, all MAC samples produced Fe_3_O_4_ and ZnO. Notably, the presence of zero-valent Fe crystals was demonstrated at 2*θ* = 44.6 and 64.9° (JCPDS 06-0696). Thus, Fe_3_O_4_, Fe^0^, and ZnO were the main products present in MAC. Compared to MBC and ZAC samples, MAC samples contained a greater amount of background noise. In MAC samples, strong activation could significantly reduce crystallinity and increase amorphous components, such as carbon base. Therefore, intense scattering may obscure the low peaks of the available crystals. In fact, several peaks of ZnO and Fe_3_O_4_ crystals in MAC samples were overlapped by background noise.

As the ZnCl_2_/LSP mass ratio rose from 0.1 to 0.4, the peak intensities of Fe_3_O_4_ declined while those of Fe^0^ increased. These results demonstrate that higher ZnCl_2_-loading content could enhance the decomposition of LSP into carbon base and H_2_O, and more ZnO could be formed. As shown in Table 1, Zn content increased from 2.24 to 3.94 wt% when the ZnCl_2_/LSP mass ratio increased from 0.1 to 0.4. Faster carbonization boosted by ZnCl_2_ could provide more decomposition products (e.g., C and H_2_O) to accelerate equations (4)–(9). As a consequence, the Fe content in MAC samples (6.89–6.94 wt%) was generally higher than that in MBC (5.69 wt%). With MBC, unreacted FeCl_3_ was eliminated via washing with distilled water. In contrast, the similar Fe content in MAC samples reveals that nearly all FeCl_3_ may be converted into Fe_3_O_4_ and Fe^0^, which were embedded in the carbon matrix. Moreover, the reduction of Fe_3_O_4_ to Fe was enhanced. Together with ZnCl_2_, this reaction could activate the porous carbon system. In fact, as the ZnCl_2_/LSP mass ratio increased, the pyrolysis efficiency fell marginally (Table 1). Despite the higher Zn-loading content, the stronger activation might reduce the remaining carbon content in MAC.

#### 3.1.2. Porous Properties of BC, MBC, and MAC

As presented in Table 1, *S*_BET_ and *V*_total_ of MBC were 274 m^2^/g and 0.14 cm^3^/g, respectively. These results are similar to previous studies for LSP-derived MBC [19, 27]. With ZnCl_2_ activation alone, *S*_BET_ and *V*_total_ of ZAC were 369 m^2^/g and 0.21 cm^3^/g, respectively. The combination of FeCl_3_ and ZnCl_2_ was therefore expected to strongly enhance the porous properties of MAC. As a result, when the ZnCl_2_/LSP mass ratio increased from 0.1 to 0.4, S_BET_ of MAC gradually rose from 531 to 1080 m^2^/g, which was 1.9–3.9 and 1.4–2.9 times more than that of MBC and ZAC, respectively. Similarly, *V*_total_ of MAC samples was 0.31–0.51 cm^3^/g, which was 2.2–3.6 and 1.5–2.4 times higher than that of MBC and ZAC, respectively. These results demonstrate that the combination of ZnCl_2_ and FeCl_3_ improved the porous carbon system remarkably.

**Figure 2:**
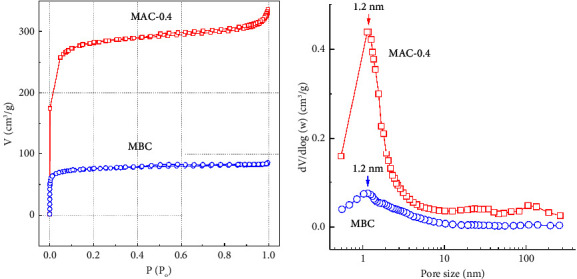
(a) Nitrogen adsorption and desorption isotherms and (b) pore size distribution of MBC and MAC-0.4.

Figure 2(a) displays the nitrogen adsorption and desorption isotherms for MBC and MAC-0.4. Extremely slim hysteresis loops resulting from capillary condensation indicated that few mesopores were formed. As a result, MBC and MAC were composed primarily of micropores. Indeed, BJH pore size distribution revealed that both the MBC and MAC-0.4 samples contained predominant micropores with similar typical pore sizes of around 1.2 nm (Figure 2(b)). Nonetheless, MAC-0.4 contained slightly more mesopores and macropores than MBC. Based on these findings, coactivation led to a significant increase in the number of micropores and a moderate enlargement in pore size inside the carbon structure.

#### 3.1.3. Magnetic Properties of MBC and MAC

**Figure 3:**
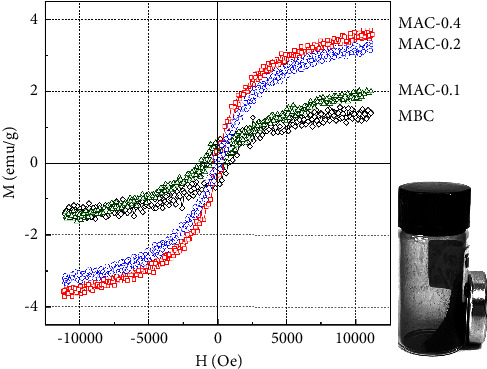
Vibrating sample magnetometer of MBC and MAC samples.

All MBC and MAC samples were easily attracted by an external magnetic field from a magnet, as illustrated in Figure 3. Furthermore, VSM investigated their magnetic properties in depth. In general, all samples displayed similar magnetic hysteresis curves with extremely low coercivity, which was indicative of superparamagnetic behavior. Consequently, these materials may be magnetized and demagnetized simply. Similar trends have been uncovered in prior research [69, 70]. In particular, MBC possessed a saturation magnetization of approximately 1.4 emu/g. The saturation magnetizations of MAC-0.1, MAC-0.2, and MAC-0.4 were 1.9, 3.3, and 3.6 emu/g, which were 1.4, 2.4, and 2.6 times that of MBC, respectively. These results indicate that coactivation could enhance the magnetic properties of the obtained MAC. As presented in Table 1, the Fe content in all MAC samples (6.89–6.94 wt%) was not much higher than that in MBC (5.69 wt%). However, Fe_3_O_4_ was predominant in MBC, whereas Fe_3_O_4_ and Fe^0^ coexisted in MAC samples. As previously discussed, when the ZnCl_2_/LSP mass ratio increased from 0.1 to 0.4, more Fe^0^ crystals were formed. Consequently, the magnetic nature of different Fe-based materials may be the primary reason for the variation in the magnetic properties of MBC and MAC. According to Feng et al. [71], when Fe_3_O_4_ was reduced to Fe, the magnetic properties of the resulting material increased because Fe can possess stronger magnetic properties than Fe_3_O_4_. In addition, other factors, such as the size, shape, magnetic anisotropy, and crystallinity of Fe_3_O_4_ and Fe^0^, which strongly depend on the preparation conditions, could influence their magnetic properties [72, 73].

#### 3.1.4. FTIR Spectroscopy of MAC

**Figure 4:**
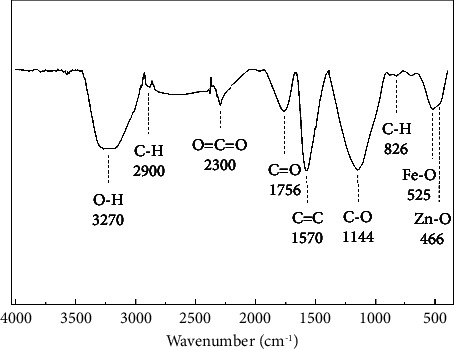
FTIR spectroscopy of MAC-0.4.

FTIR spectroscopy of MAC-0.4 is presented in Figure 4. Different peaks were found in MAC, including 3270 cm^−1^ (O-H stretching vibrations), 2900 cm^−1^ (C-H stretching vibrations), 2300 cm^−1^ (O=C=O stretching vibrations), 1756 cm^−1^ (C=O stretching vibrations), 1570 cm^−1^ (C=C stretching vibrations), 1144 cm^−1^ (C-O stretching vibrations), and 826 cm^−1^ (C-H stretching vibrations) [74–76]. Notably, peaks at 525 cm^−1^ could be Fe-O bonds [77, 78], and 466 cm^−1^ could be Zn-O bonds [41, 79]. More importantly, the presence of polar oxygen-rich functional groups on the surface of MAC-0.4 could improve its interaction with organic pollutants and oxidizing agents during catalytic treatment processes.

#### 3.1.5. SEM Images of MAC

**Figure 5:**
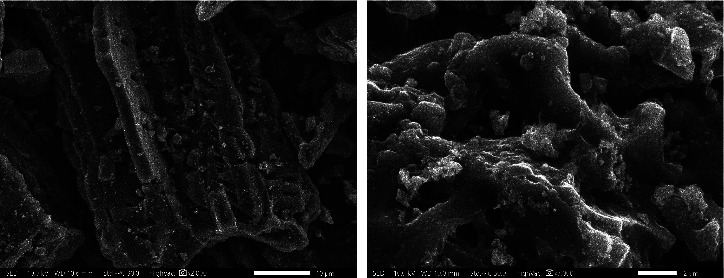
SEM images of MAC-0.4.

The surface morphology of MAC-0.4 was observed by SEM images (Figure 5). Sharp-edged fragments could be generated from vigorously crushing LSP. In addition, such macropores at the microscale were found. Depending on the porous properties, ZnCl_2_ and FeCl_3_ coactivation of LSP might affect micropores more than mesopores and macropores. Therefore, those macropores could come from the natural vascular bundles of LSP [80, 81]. Especially, it seems that few Fe- and Zn-based particles were observed. These components may be embedded in the carbon framework without forming clusters on the MAC surface. This finding is similar to that of MBC in previous studies [27, 28]. Of particular importance, the firm immobilization is anticipated to enhance the stability and reusability of the MAC catalyst.

#### 3.1.6. EDX Spectroscopy and Elemental Mapping of MAC

**Figure 6:**
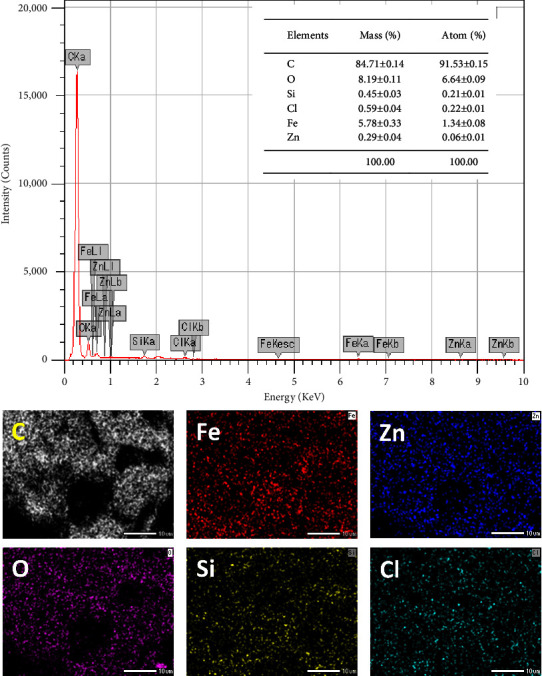
EDX spectroscopy and elemental mapping of MAC-0.4.

EDX spectroscopy and elemental mapping were used to determine the chemical composition and elemental distribution on the surface of MAC-0.4 (Figure 6). The predominant elements included C (84.71 wt%), Fe (5.78 wt%), and O (8.19 wt%). Notably, the surface Fe content detected by EDX was close to the bulk Fe content analyzed by AAS (6.89 wt%). The EDX result may show the surface distribution of Fe, whereas the AAS analysis may give the bulk Fe content (both outside and inside the carbon matrix). In traditional methods, the Fe element is normally decorated on the surface of the carbon base rather than inside the carbon framework. As a result, the surface Fe content from EDX may be much higher than the bulk Fe content from AAS. Herein, FeCl_3_ was impregnated inside LSP. Hence, the distribution of Fe may be spread throughout the carbon structure, resulting in comparable Fe contents from EDX and AAS results. Unlike Fe, the minor surface Zn content (0.29 wt%) was much lower than the bulk Zn content (3.94 wt%). It reveals that Zn on the carbon surface may readily be removed during pyrolysis. As previously indicated, ZnCl_2_ vapor could be formed and diffused into porous carbon. Due to its high mobility, ZnCl_2_ vapor may escape off the MAC surface and be carried away by the flow of N_2_ gas. Then, only the inner carbon matrix may retain Zn better. Interestingly, the atomic ratio of O/Fe was approximately 5.0, which is much higher than that of Fe_3_O_4_. This comparison demonstrates that a considerable surface O content was present in the functional groups, as listed in the FTIR results.

For the remaining elements in MAC, Si and Cl were identified at 0.45 and 0.59 wt%, respectively. Several reports demonstrate that minor elements, including Si, can be present in LSP [82, 83]. However, Cl may be partially or entirely derived from the additional FeCl_3_ and ZnCl_2_. As previously stated, MAC was cleaned until no Fe^3+^, Zn^2+^, or Cl^−^ leaching was detected. Therefore, these elements could be firmly bound within the carbon matrix by strong mechanical or chemical linkages [27]. Lastly, element mapping showed that Fe, Zn, O, Cl, and Si elements were uniformly distributed on the carbon surface at the microscale. The consistent spread of Fe and Zn may be a result of well-loaded FeCl_3_ and ZnCl_2_ in LSP. Following is a discussion on nanoscale TEM analysis for clarifying the interior structure of materials.

#### 3.1.7. TEM Images of BC, MBC, and MAC

**Figure 7:**
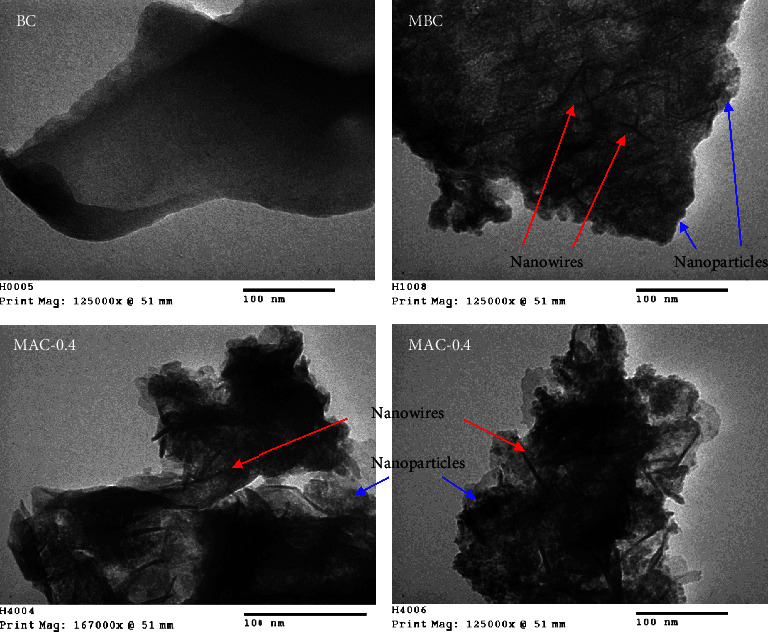
TEM images of BC, MBC, and MAC-0.4.

TEM images were used to observe the internal structures of BC, MBC, and MAC-0.4 (Figure 7). BC shows a smooth surface with a gradual transition in brightness. Contrarily, the inconsistent brightness in MBC reveals the morphology of Fe_3_O_4_. At the nanoscale, dust-like Fe_3_O_4_ particles were observed throughout the carbon matrix. These nanoparticles seem to group together in clusters. In addition to nanoparticles, MBC contained nanowires of Fe_3_O_4_. Intriguingly, the existence of magnetic nanowires in MBC is rare. It appears possible that Fe_3_O_4_ nanowires may be formed in nanopores that resemble tubes [27]. Similar to MBC, MAC-0.4 had nanoparticles and nanowires that were well distributed throughout the carbon matrix. However, not only Fe_3_O_4_ but also Fe^0^ and ZnO crystals were present in MAC. It was suggested that the initial natural porous structure of LSP for ZnCl_2_ and FeCl_3_ loading played an important role in the morphology of Fe- and Zn-based products. LSP contains natural cellulose fibers [84]. Wire-like morphology may, therefore, result from crystallization in an extremely narrow fibrous matrix. More importantly, well-distributed Fe-based components at the nanoscale in the porous carbon system of MAC could not only improve its catalytic stability but also provide a greater contact area with other species for higher catalytic activity [78]. These advantages were explored in Fenton-like catalysis for the degradation of acid orange 10.

### 3.2. Removal of Acid Orange 10 Using Magnetic Activated Carbon

#### 3.2.1. MAC as an Adsorbent for AO10 Removal

**Figure 8:**
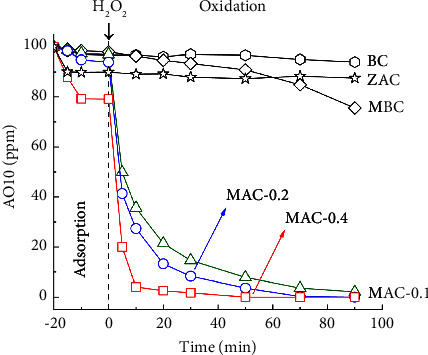
AO10 degradation by BC, ZAC, MBC, and MAC catalysts (0.20 g/L catalyst, 350 ppm H_2_O_2_, 100 ppm AO10, and pH 3.0).

**Figure 9:**
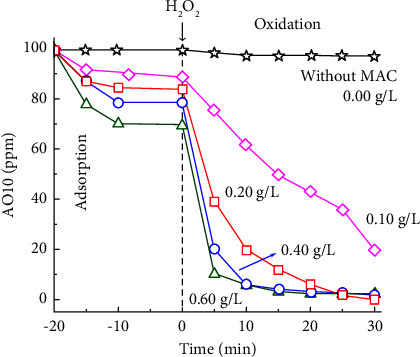
Effects of MAC dosage on AO10 degradation (MAC-0.4, 350 ppm H_2_O_2_, 100 ppm AO10, and pH 3.0).

**Figure 10:**
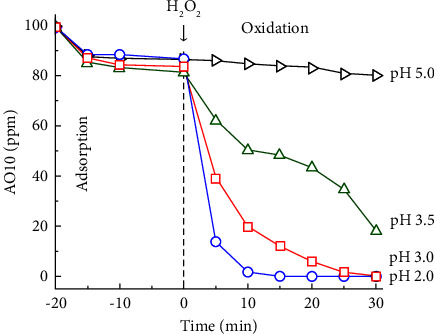
Effects of pH on AO10 degradation catalyzed by MAC (0.20 g/L MAC-0.4, 350 ppm H_2_O_2_, and 100 ppm AO10).

**Figure 11:**
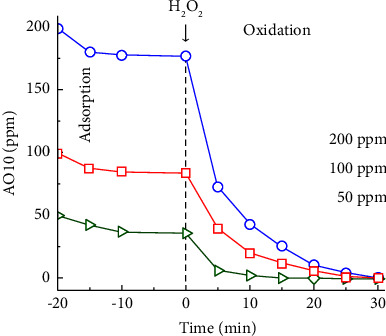
Degradation of different AO10 concentrations by MAC catalyst (0.20 g/L MAC-0.4, *C*_*H*_2_*O*_2__/*C*_*AO*10_ = 3.5, and pH 3.0).

**Figure 12:**
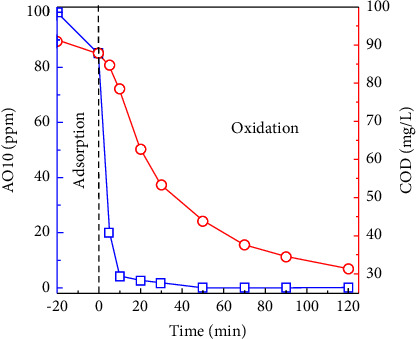
Changes in AO10 and COD concentrations during Fenton-like degradation catalyzed by MAC (0.20 g/L MAC-0.4, 350 ppm H_2_O_2_, and pH 3.0).

**Figure 13:**
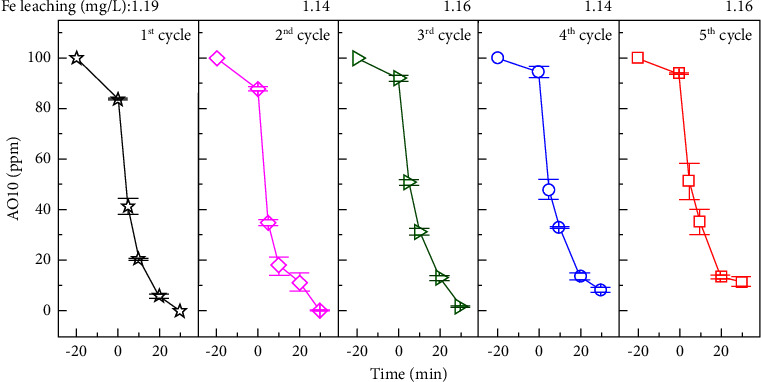
Stability and reusability of MAC for AO10 degradation (0.20 g/L MAC-0.4, 350 ppm H_2_O_2_, and pH 3.0).

The catalytic activity of MAC in AO10 degradation using H_2_O_2_ was investigated. For complete oxidation of 100 ppm AO10, a minimum of 316 ppm H_2_O_2_ is theoretically required [44]. According to Do et al. [27], 350 ppm was an appropriate dosage for the degradation of 100 ppm AO10. Thus, that dosage was selected. In addition to MAC, BC, ZAC, and MBC were used as blank samples. Because BC, ZAC, MBC, and MAC samples could potentially adsorb a certain amount of AO10, the experiments were divided into two stages: adsorption for the first 20 min, followed by 30 min of oxidation (Figures 8–13). Parameters, including MAC catalysts, MAC dosage, pH, and AO10 concentration, are presented in Table 2. All results revealed that the adsorption process closely reached equilibrium within 20 min before the next oxidation step. Although the experimental parameters were designed for catalytic oxidation, MAC exhibited excellent adsorption performance for AO10. In actuality, low MAC-0.4 dosages (0.10 to 0.40 g/L) eliminated AO10 with adsorption capacities ranging from 49.9 to 106.0 mg/g. Moreover, these quantities were much higher than those of BC, MBC, and ZAC. These results indicate that porous carbon systems (*S*_BET_ and *V*_total_) in carbon-based materials could play an important role in AO10 removal. Furthermore, *π*-*π*, hydrogen, and electrostatic interactions between functional groups on the MAC surface and AO10 [23, 85] may aid in effective adsorption processes.

#### 3.2.2. Effects of MAC Prepared by Different ZnCl_2_/LSP Mass Ratios on AO10 Degradation

AO10 degradation was carried out with BC, ZAC, MBC, and MAC catalysts, as shown in Figure 8. BC removed a small amount of AO10, mainly by adsorption. With ZnCl_2_ activation, ZAC eliminated 10.0% of AO10 through adsorption, and then almost lacked catalytic AO10 degradation in the subsequent step. Conversely, all MBC and MAC catalysts showed certain catalytic activity toward AO10 degradation. These results indicated that Fe-based components rather than ZnO and carbon-based support were the active sites for these catalytic processes. The MBC sample contained Fe_3_O_4_ crystals, while the MAC samples contained both Fe_3_O_4_ and Fe^0^ crystals. These Fe sites could catalyze AO10 degradation as follows (C− denotes that the Fe sites were incorporated into the carbon matrix) [77]:

(12) $$\begin{array}{c} C-{Fe}^{0}+H_{2}O_{2}+2H^{+}⟶\;C-{Fe}^{II}+2H_{2}O \end{array}$$

(13) $$\begin{array}{c} C-{Fe}^{II}+H_{2}O_{2}\;⟶\;C-{Fe}^{III}+•OH+{OH}^{-} \end{array}$$

(14) $$\begin{array}{c} C-{Fe}^{III}{+H}_{2}O_{2}\;⟶\;C-{Fe}^{II}+H^{+}+•OOH \end{array}$$

(15) $$\begin{array}{c} Acid\;orange\;10+•OH\;⟶\;Intermediates\;⟶\;Mineralization \end{array}$$

Compared with MBC, all MAC samples showed much faster AO10 decolorization rates. In addition, increasing the ZnCl_2_/LSP mass ratio improved the decolorization rate of AO10. As presented in Table 2, the Fe content in different MAC samples (6.89–6.94 wt%) was not much different and slightly higher than that in MBC (5.69 wt%). It reveals that other parameters, such as the nature, distribution, shape, and size of Fe-based crystals, may affect the catalytic activity of MBC and MAC. Several reports found that the composite of Fe^0^ and Fe_3_O_4_ exhibited higher catalytic performance than each component [86, 87]. The galvanic cell formed between Fe^0^ and Fe_3_O_4_ may facilitate electron transfer and •OH generation. MAC contained not only Fe^0^ but also Fe_3_O_4_, which may follow this synergic effect. Furthermore, Fe-based components were fixed in the carbon supports, which could affect the process indirectly. MBC and MAC samples had different porous properties (*S*_BET_ and *V*_total_) and crystal structures of Fe-based components. As mentioned before, the nanoscale Fe sites were well dispersed in the porous carbon system of MAC-0.4, which had a high *S*_BET_ and a large *V*_total_. Hence, mass transfer in these pores might become more convenient, and more catalytic sites with high residual energy might be accessible. These main advantages might explain the robust enhancement of the catalytic oxidation of AO10 by H_2_O_2_.

The presence of minor elements in MAC might impact its catalytic activity. According to such reports, Cl^−^ ions could be detrimental to AO10 degradation [88, 89]. The inhibitory effect of Cl^−^ ions may be a result of their interaction with •OH. However, MAC was carefully rinsed to remove all water-soluble components. Consequently, trace quantities of Cl and Si may not exist as ions or be firmly bound within the carbon framework. It may be challenging to leach those elements into the treatment media. Due to the strong catalytic activity of MAC on AO10 degradation, the significance of these trace elements may be negligible.

#### 3.2.3. Effects of MAC Dosage on AO10 Degradation

Figure 9 depicts the relationship between MAC dosage and AO10 degradation. Without a catalyst, it was nearly impossible for H_2_O_2_ to eliminate AO10. In contrast, when MAC was applied, AO10 decolorization occurred rapidly. With MAC dosages between 0.20 and 0.60 g/L, AO10 was nearly completely decolored within 30 min. These results demonstrated that MAC catalyzed this decolorization effectively. In addition, the decolorization rate generally increased when the MAC dosage rose from 0.10 to 0.60 g/L. A high catalyst dosage may increase the number of active sites for H_2_O_2_ decomposition into •OH radicals. However, increasing the MAC dosage from 0.40 to 0.60 g/L did not result in a significant improvement. According to reports in the literature, the excess catalyst might deactivate the originally generated •OH radicals, as shown in the following equation [90-91]:

(16) $$\begin{array}{c} C-Fe^{II}+•OH\;⟶\;C-Fe^{III}+{OH}^{-} \end{array}$$

#### 3.2.4. Effects of pH on AO10 Degradation Catalyzed by MAC

pH can be a crucial variable for AO10 degradation catalyzed by MAC on the basis of the Fenton-like mechanism. As shown in Figure 10, AO10 degradation in the pH range of 2.0–5.0 was investigated. The AO10 concentration almost remained constant at pH 5.0. In high pH conditions, Fe(OH)_3_ can be formed from Fe(III) and cover the active sites of the catalyst, making H_2_O_2_ adsorbed and reducing the amount of free •OH radicals formed [92, 93]. Nonetheless, AO10 decolorization occurred rapidly at low pH. In general, when pH decreased from 3.5 to 2.0, the AO10 decolorization rate increased remarkably. At pH 2.0, the AO10 decolorization nearly finished within 15 min, while that at pH 3.0 required 30 min. In acidic conditions, the transition between Fe(II) and Fe(III) could become convenient. As a result, a large number of •OH radicals could be produced rapidly. These results were consistent with other Fenton-like research in the literature. Although pH 2.0 offered robust and complete AY23 decolorization within 30 min of H_2_O_2_ addition, low pH could promote Fe leaching, leading to a homogeneous mechanism for catalytic decolorization [93]. Overall, pH 3.0 may be appropriate for achieving a heterogeneous mechanism and maintaining high decolorization efficiency.

#### 3.2.5. Degradation of Different AO10 Concentrations by MAC Catalyst

One of the important factors influencing degradation efficiency is the concentration of pollutants. Therefore, AO10 degradation at different concentrations of 50–200 ppm was explored. As discussed before, 100 ppm of AO10 was effectively decolorized by 350 ppm of H_2_O_2_. In the same H_2_O_2_/AO10 mass ratio, 50 and 200 ppm of AO10 were investigated with 175 and 700 ppm of H_2_O_2_, respectively. Although the MAC dosage was kept at 0.20 g/L, AO10 was completely eliminated within 30 min of oxidation (Figure 11). As presented in Table 3, the average decolorization rates in 30 min at 100 and 200 ppm AO10 were 16.7 and 33.3 mg AO10/g MAC/min, respectively. These results demonstrated that MAC could catalyze AO10 degradation effectively over a wide concentration range.

The Fenton-like catalytic performance of MAC for AO10 degradation was compared with that of other catalysts (Table 3). In general, most catalysts require long treatment times and high catalyst dosages at low AO10 concentrations. In a previous study, LSP-derived MBC showed good catalytic activity for AO10 degradation, with almost all AO10 being decolorized within 90 min [27]. Its average decolorization rate was 2.8 mg AO10/g MBC/min, which was much higher than that of other catalysts. However, at a similar condition, the average decolorization rate catalyzed by MAC was 6.0-fold higher than that by MBC. These comparisons prove that the catalytic performance of MAC is superior to that of other catalysts. As discussed before, well-dispersed Fe-based nanocrystals in the porous carbon system with high *S*_BET_ and *V*_total_ and the synergic effect of Fe^0^ and Fe_3_O_4_ might explain the robust enhancement of the catalytic oxidation of AO10 by H_2_O_2_.

#### 3.2.6. COD Reduction during AO10 Degradation Catalyzed by MAC

COD is defined as the total amount of oxygen required for the oxidation of organic matter into CO_2_ and H_2_O [97]. It is an important parameter to determine the degree of mineralization during the treatment of organic compounds and is subject to strict regulation by environmental regulatory agencies [98]. Here, changes in COD and AO10 concentrations during Fenton-like degradation catalyzed by MAC were carried out (Figure 12). At the beginning, 100 ppm AO10 provided 91 mg/L COD. In the initial adsorption step, AO10 and COD concentrations were lowered in part. With the MAC-catalyzed acceleration, the AO10 concentration in the subsequent oxidation process fell rapidly and nearly complete in 30 min. At the same time, COD declined gradually from 88 to 53 mg/L, and this tendency continued throughout the later period. At 120 min, COD dropped to 31 mg/L, corresponding to 66% of COD elimination. Thus, despite the fact that AO10 was decolorized during the first period, certain organic intermediates might require additional time to be completely mineralized [99, 100]. Unselectively, reactive •OH radicals can attack species. As a result, AO10 can be converted into numerous intermediates like aniline, phenol, 7-hydroxy-8-(hydroxyamino) naphthalene-1,3-disulfonic acid, 7,8-dihydroxy-naphthalene-1,3-disulfonic acid, alpha naphthol, and carboxylic acid. To completely mineralize AO10, additional treatment time may be necessary, or the Fenton-like process can be combined with other treatments [27, 96].

#### 3.2.7. Stability and Reusability of MAC Catalyst

Catalyst stability and reusability play crucial roles in industrial pollutant remediation. In order to investigate those characteristics of the MAC catalyst, five consecutive cycles of AO10 degradation were performed in triplicate.  Figure 13 depicts the mean values for the experiments. After 20 min of adsorption, H_2_O_2_ was added to each cycle, and the treated solution was analyzed for Fe leaching. As a result, the catalytic performance of MAC-0.4 remained effective even after five cycles. At 20 min of oxidation, the total AO10 removal after each cycle was 93.9 ± 0.9%, 88.4 ± 3.5%, 86.9 ± 0.9%, 86.2 ± 1.4%, and 86.3 ± 0.8%, respectively. The removal decreased slightly in the second cycle, then stabilized in the subsequent three cycles. It appears that unstable Fe sites were leaked into the treated medium in the first step. The remaining Fe sites in a recycled catalyst may be firmly anchored in the MAC framework and offer stable catalytic performance in the following cycles. Furthermore, the adsorption capacity of MAC on AO10 decreased with each cycle. It seems that distilled water and ethanol cannot eliminate adsorbates entirely. Consequently, it may impact the catalytic performance of the used MAC-0.4 in the subsequent cycle. Lastly, AAS results revealed that 1.14–1.19 mg/L of Fe leaching was detected after each cycle. This leaching was below the limit concentration of 2 mg/L established by European Union directives for treated water.

## 4. Conclusion

In summary, magnetic activated carbon was successfully prepared using one-pot pyrolysis of ZnCl_2_ and FeCl_3_-loaded lotus seedpod waste. The as-prepared MAC had a high *S*_BET_ of 1080 m^2^/g, a large *V*_total_ of 0.51 cm^3^/g, and a strong saturation magnetization of 3.6 emu/g, which were 3.9-fold, 3.6-fold, and 1.8-fold higher than those of MBC. With 6.89 wt% Fe and 3.94 wt% Zn, different crystals of Fe_3_O_4_, Fe^0^, and ZnO were present in MAC. Interestingly, TEM images showed that their nanoparticles and nanowires were developed inside the carbon matrix. Subsequently, MAC was investigated for the treatment of acid orange 10. As a result, MAC demonstrated both a useful adsorbent and an efficient Fenton-like catalyst. At pH 3.0, 0.20 g/L MAC removed AO10 (100 ppm) with an adsorption capacity of 78.4 mg/g. When 350 ppm of H_2_O_2_ was added, AO10 decolorization occurred rapidly and was practically complete within 30 min. At 120 min, 66% of the COD was removed. Moreover, the catalytic performance remained stable, with total AO10 removal slightly decreasing from 93.9 ± 0.9% to 86.3 ± 0.8% after five consecutive cycles. The minimal iron leaching ranged from 1.14 to 1.19 mg/L. In conclusion, these results indicated that magnetic activated carbon derived from ZnCl_2_ and FeCl_3_ coactivation of lotus seedpod residue is an efficient catalyst for robust acid orange 10 decolorization.

## Figures and Tables

**Table 1:** Properties of MBC, ZAC, and MAC prepared with different ZnCl_2_/FeCl_3_/LSP mass ratios.

Materials	ZnCl_2_/FeCl_3_/LSP (w/w/w)	Pyrolysis efficiency (%)	Fe (wt%)	Zn (wt%)	*S*_BET_(m^2^/g)	*V*_total_(cm^3^/g)	*d*_average_ (nm)	Saturation magnetization (emg/g)
ZAC	0.4/−/1.0	—	—	—	369	0.21	1.2	—
MBC	−/0.2/1.0	49.9 ± 0.3	5.69	—	274	0.14	2.0	1.4
MAC-0.1	0.1/0.2/1.0	53.7 ± 0.3	6.94	2.24	531	0.31	2.4	1.9
MAC-0.2	0.2/0.2/1.0	51.2 ± 0.3	6.92	2.49	780	0.46	2.4	3.3
MAC-0.4	0.4/0.2/1.0	49.6 ± 0.2	6.89	3.94	1080	0.51	1.9	3.6

**Table 2:** Adsorption and catalytic degradation of acid orange 10 by BC, MBC, and MAC samples.

Samples	Catalyst (g/L)	pH	AO10 (ppm)	H_2_O_2_ (ppm)	Adsorption (after 20 min)		Oxidation (after 30 min)		
					AO10 (ppm)	Adsorption capacity (mg/g)	AO10 (ppm)	Efficiency (%)	Total removal (%)
BC	0.20	3.0	100	350	96.6	16.6	96.1	0.7	3.9
ZAC					90.0	50.0	87.3	3.0	12.7
MBC					98.1	9.5	88.6	9.6	11.4
MAC-0.1					97.0	14.9	14.6	84.9	85.4
MAC-0.2					93.9	30.3	8.3	91.2	91.7
MAC-0.4					84.3	78.4	n.d.	100	100

MAC-0.4	0.10	3.0	100	350	89.4	106.0	20.0	77.6	80.0
	0.20				84.3	78.4	n.d.	100	100
	0.40				79.1	52.2	1.6	98.0	98.4
	0.60				70.1	49.9	1.8	97.4	98.2

MAC-0.4	0.20	2.0	100	350	87.5	62.7	n.d.	100	100
		3.0			84.3	78.4	n.d.	100	100
		3.5			82.0	90.0	18.2	77.8	81.8
		5.0			86.7	66.5	80.6	7.0	19.4

MAC-0.4	0.20	3.0	50	175	36.1	69.6	n.d.	100	100
			100	350	84.3	78.4	n.d.	100	100
			200	700	178.0	90.0	0.3	99.8	99.7

n.d.: not detected.

**Table 3:** Comparison of catalytic activity of MAC and other catalysts for AO10 degradation.

Catalyst	pH	AO10 (ppm)	H_2_O_2_(ppm)	Catalyst (g/L)	Time (min)	Decolorization efficiency (%)	Average decolorization rate (mg/g/min)	Reference
*α*-FeOOH	3.0	50	1000	1.0	180	99.6	0.3	[94]
Nano Fe^0^	3.0	40	20	0.7	120	93.7	0.4	[95]
Fe/biochar	3.0	100	75	0.5	300	99.7	0.7	[43]
Nano Fe_3_O_4_/CeO_2_	2.5	50	1020	2.0	120	98.2	0.2	[96]
MBC	3.0	100	350	0.4	90	100.0	2.8	[27]
MAC	3.0	100	350	0.2	30	100.0	16.7	This work
MAC	3.0	200	700	0.2	30	99.8	33.3	This work

## Data Availability

No new data were created or analyzed in this study.
